# Diverse Changes of Circulating Fibroblast Growth Factor 21 Levels in Hepatitis B Virus-Related Diseases

**DOI:** 10.1038/s41598-017-16312-6

**Published:** 2017-11-28

**Authors:** Liang Wu, Qingchun Pan, Guangyu Wu, Lingling Qian, Jing Zhang, Lei Zhang, Qichen Fang, Guoqing Zang, Yudong Wang, George Lau, Huating Li, Weiping Jia

**Affiliations:** 1 Department of Endocrinology and Metabolism, Shanghai Jiao Tong University Affiliated Sixth People’s Hospital; Shanghai Diabetes Institute; Shanghai Clinical Center for Diabetes, Shanghai, China; 2 Department of Medicine, Shanghai Jiao Tong University School of Medicine, Shanghai, China; 3 Department of Infectious Diseases, Shanghai Jiao Tong University Affiliated Sixth People’s Hospital, Shanghai, China; 4 Division of Gastroenterology and Hepatology, Humanity and Health Medical Centre, Hong Kong S.A.R, China; 5 Second Liver Cirrhosis Diagnosis and Treatment Center, Beijing 302 Hospital, Beijing, China; 6 Institute of Translational Hepatology, Beijing 302 Hospital, Beijing, China

## Abstract

Fibroblast growth factor 21 (FGF21), a stress-induced hormone in the liver, has been shown the protective functions in pathological conditions. The study investigated the association of circulating FGF21 with hepatitis B virus (HBV) infection and its related diseases. Serum FGF21 levels were measured in 33 acute hepatitis B (AHB), 75 chronic hepatitis B (CHB) and 66 CHB patients with advanced liver diseases including liver cirrhosis, acute-on-chronic liver failure (ALCF) and hepatocellular carcinoma (HCC) together with 200 age- and BMI-matched healthy controls. FGF21 levels were significantly increased in AHB patients and rapidly returned to normal levels after treatment. FGF21 levels reflected the degree of liver injury caused by AHB. However, serum FGF21 levels were decreased in CHB patients especially in those who developed cirrhosis and were associated with hepatic protein synthesis capacity. Serum FGF21 in CHB patients were increased with the occurrence of ACLF. Notably, in CHB patients who developed HCC, serum FGF21 exhibited a dramatic increase, which may provide important information on monitoring tumorigenesis in CHB patients. In conclusion, we revealed the diverse changes of circulating FGF21 in HBV-related diseases. FGF21 may be a useful biomarker in monitoring the tumorigenesis in patients with CHB.

## Introduction

Hepatitis B virus (HBV) is the most common hepatitis virus with more than two billion people have been infected worldwide, and it is estimated that 50 million new cases are added every year and 240 million people are chronically infected with hepatitis B, most in Asia and Africa^1,2^. Chronic hepatitis B (CHB) infection may progress to liver cirrhosis and liver failure, and is the major cause of hepatocellular carcinoma (HCC)^3^. Monitoring the disease progression of HBV infection has been a major clinical concern for the early treatment of associated liver disease and lowering morbidity and mortality. Traditional biomarkers like alanine aminotransferase (ALT) have been widely used for detecting liver injury^4^. When liver injury occurs, the enzymes that are aggregated in the cytosol of the hepatocytes are released into circulation and cause an elevation in serum concentration^5^. Notably, hepatokines that response to liver stress may reflect liver status, and provide a noninvasive and easy-to-perform means of monitoring therapeutic effect and progression in HBV-related diseases.

Fibroblast growth factor 21 (FGF21) is an important metabolic regulator that is predominantly produced by the liver^6,7^. Despite its pleiotropic metabolic effects on maintaining glucose and lipid homeostasis, growing evidence from both animal and clinical studies has demonstrated that FGF21 is involved in multiple liver stimuli. FGF21 expression is significantly induced in hepatocytes in response to carcinogenic transformation in mice undergoing chemical and genetic-induced hepatocarcinogenesis^8^. Acetaminophen overdose causes a drastic increase in both circulating levels and hepatic expression of FGF21 in mice, which in turn acts as a feedback signal to protect mice from acetaminophen-induced hepatotoxicity by enhancing the antioxidant capacity^9^. In humans, serum FGF21 levels are elevated in the early phase of patients with nonalcoholic fatty liver disease (NAFLD) and positively correlates with gamma-glutamyl transpeptidase (GGT)^10^. Moreover, in patients with liver transplantation, serum FGF21 levels rise sharply and reach the peak as early as two hours after reperfusion, whereas the peak of serum ALT levels appears at 24 hours after reperfusion, suggesting a superior sensitivity of serum FGF21 compared to the currently used biomarkers for detection of hepatic ischemia/reperfusion injury^11^.

FGF21 may reflect functional status of the liver, but the role of FGF21 in hepatitis viral infection is still unclear. A previous study performed in 75 chronic hepatitis C patients showed that serum FGF21 were significantly higher in those subjects compared with those in healthy subjects^12^. However, another study demonstrated that the elevation of hepatitis C virus RNA was accompanied by a decreased expression of FGF21 mRNA in the liver^13^. Chronic HBV infection may progress to HBV-related liver diseases including cirrhosis, acute-on-chronic liver failure (ACLF) and HCC^3^, but the association between hepatic FGF21 expression and the progression of those diseases is largely unknown. The objective of the study was to investigate whether serum FGF21 levels are altered in HBV-infected patients and to explore the association of serum FGF21 levels with HBV-related diseases.

## Results

### Serum FGF21 levels were increased in AHB patients but decreased in CHB patients

The clinical and laboratory characteristics of the patients with AHB and CHB are summarized in Table 1. Compared with controls, patients with AHB and CHB had similar age and BMI. Biochemical indicators of liver injury were significantly higher in AHB patients than those in controls, including ALT, aspartate aminotransferase (AST), GGT, alkaline phosphatase (ALP), total bilirubin (TBil), total bile acid (TBA) and c-reactive protein (CRP) (all P < 0.001). Serum FGF21 levels were found significantly higher in AHB patients (243.3 pg/ml [189.6–451.7]) than those in control subjects (198.4 pg/ml [152.8–272.2]) (P < 0.001). After antiviral and supportive treatment for 11.8 ± 1.7 days, serum FGF21 levels in AHB patients were significantly decreased (112.8 pg/ml [83.2–186.3], P = 0.001) together with the indicators of liver injury.

**Table 1:** Anthropometric and biochemical parameters in control, AHB, AHB after treatment and CHB groups.

Variables	Control (n = 200)	AHB (n = 33)	AHB after treatment (n = 12)	CHB (n = 75)
Age (year)	40.4 ± 9.5	44.7 ± 14.9^*^	35.9 ± 11.8	35.9 ± 11.7^*#^
Male (n, %)	67 (34%)	27 (82%)	8 (67%)	55 (73%)
BMI (kg/m^2^)	22.1 ± 2.2	22.0 ± 2.2	20.2 ± 2.4^*,#^	22.3 ± 2.8
AST (U/L)^†^	18.0 (16.0, 21.0)	690.0 (351.5, 845.0)^*^	54.0 (26.3, 120.5)^*,#^	157.0 (79.0, 315.0)^*,#^
ALT (U/L)^†^	14.0 (10.0, 18.0)	1548.0 (1205.0, 2141.5)^*^	299.5 (58.3, 422.8)^*,#^	320.0 (161.0, 754.0)^*,#^
GGT (U/L)^†^	16.0 (13.0, 20.0)	175.0 (136.0, 307.5)^*^	118.0 (83.0, 224.3)^*^	84.0 (50.0, 166.0)^*,#^
ALP (U/L)^†^	64.0 (53.0, 76.0)	144.0 (115.0, 175.0)^*^	84.0 (79.0, 111.3)^*,#^	86.0 (66.0, 111.0)^*,#^
TBil (μmol/L)^†^	11.2 (8.5, 14.6)	74.4 (36.9, 145.4)^*^	21.3 (16.2, 26.4)^*,#^	18.5 (13.3, 41.5)^*,#^
TBA (μmol/L)^†^	3.3 (2.3, 4.8)	109.8 (30.8, 212.1)^*^	7.7 (4.9, 19.6)^*,#^	16.2 (9.1, 53.9)^*,#^
CRP (mg/L)^†^	0.4 (0.2, 0.9)	5.7 (1.9, 15.4)^*^	/	2.0 (1.6, 4.4)^*,#^
Cholinesterase (U/L)^†^	330.0 (290.3, 370.8)	255.0 (226.5, 299.5)^*^	264.0 (207.8, 316.0)^*^	268.0 (186.0, 320.0)^*^
A/G^†^	1.88 (1.72, 2.05)	1.63 (1.51, 1.78)^*^	1.81 (1.59, 2.08)	1.60 (1.40, 1.80)^*^
Albumin (g/L)^†^	48.0 (46.0, 49.0)	41.0 (38.5, 42.5)^*^	43.0 (38.5, 45.0)^*^	42.0 (38.0, 44.0)^*^
Total protein (g/L)^†^	73.0 (70.3, 76.0)	64.0 (61.0, 68.5)^*^	65.0 (62.3, 70.3)^*^	67.0 (62.0, 71.0)^*^
TC (mmol/L)^†^	4.4 (3.9, 5.0)	3.5 (3.2, 4.2)^*^	/	4.0 (3.7, 4.5)^*,#^
Triglyceride (mmol/L)^†^	1.0 (0.7, 1.4)	1.7 (1.1, 2.4)^*^	/	1.1 (0.8, 1.4)^#^
HDL-C (μmol/L)^†^	1.4 (1.2, 1.6)	0.5 (0.3, 0.8)^*^	/	1.2 (0.6, 1.4)^*,#^
LDL-C (μmol/L)^†^	2.7 (2.3, 3.3)	1.9 (1.5, 2.5)^*^	/	2.2 (1.7, 2.7)^*^
LgHBV-DNA (IU/ml)^†^	/	3.7 (1.0, 5.6)	1.0 (1.0, 2.0)^#^	6.8 (5.4, 7.6)^#^
FGF21 (pg/ml)^†^	198.4 (152.8, 272.2)	243.3 (189.6, 451.7)^*^	112.8 (83.2, 186.3)^*,#^	142.8 (82.0, 265.3)^*,#^

*P value < 0.05 vs. control. ^#^P value < 0.05 vs. AHB. ^†^Log-transformed before analysis.

CRP and lipid profiles in the group of AHB after treatment were not measured.

A/G, albumin/globulin; AHB, acute hepatitis B; ALP, alkaline phosphatase; ALT, alanine aminotransferase; AST, aspartate aminotransferase; BMI, body mass index; CRP, c-reactive protein; CHB, chronic hepatitis B; FGF21, fibroblast growth factor 21; GGT, gamma-glutamyl transpeptidase; HBV, hepatitis B virus; HDL-C, high-density lipoprotein cholesterol; LDL-C, low-density lipoprotein cholesterol; TBA, total bile acid; TBil, total bilirubin; TC, total Cholesterol.

**Figure 1:**
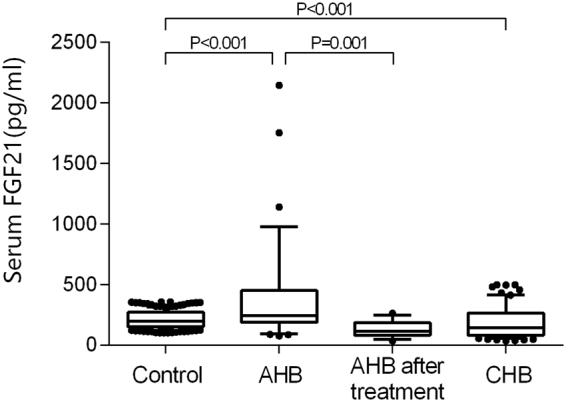
Serum FGF21 concentrations in controls and patients with AHB or CHB. Data are shown as box-and-whisker plots. The horizontal line in the middle of each box indicates the median value. The top and bottom borders of the boxes represent the 75th and 25th percentiles, respectively; the whisker represents the 10th and 90th percentiles, respectively; and the dots represent the outliers.

In patients with CHB, liver synthetic function was impaired, reflected by lower albumin, total protein, and cholinesterase (all P < 0.001). Unlike in AHB patients, serum FGF21 levels in CHB patients (142.8 pg/ml [82.0–265.3]) were significantly lower than those in controls (P < 0.001). (Fig. 1).

### Serum FGF21 levels further decreased in HBV-related liver cirrhosis but increased in CHB patients developed ACLF or HCC

**Figure 2:**
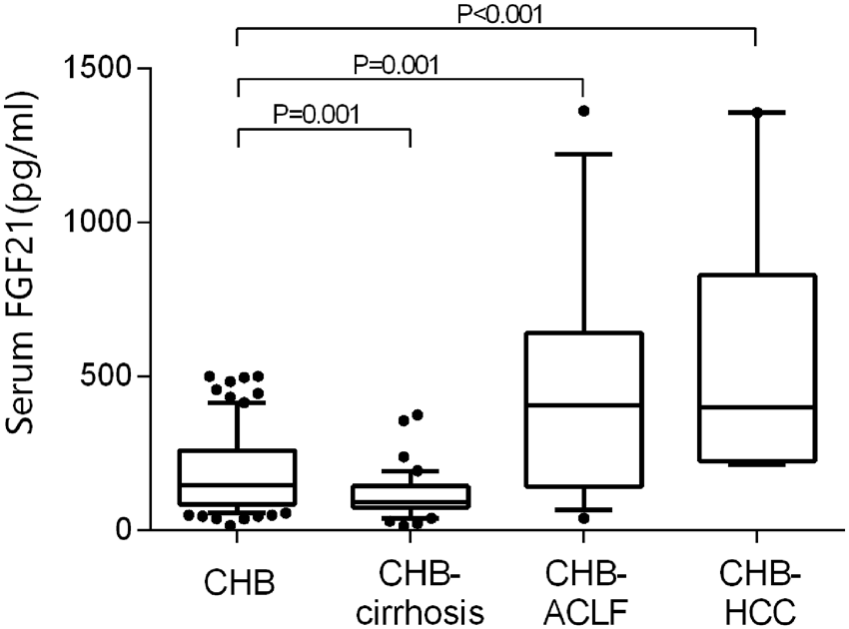
Serum FGF21 levels in patients with advanced liver diseases. Data are shown as box-and-whisker plots. The horizontal line in the middle of each box indicates the median value. The top and bottom borders of the boxes represent the 75th and 25th percentiles, respectively; the whisker represents the 10th and 90th percentiles, respectively; and the dots represent the outliers.

We next investigated the changes of serum FGF21 in chronic HBV infected patients with advanced liver diseases. Their clinical and laboratory characteristics are summarized in Table 2. Compared with CHB patients, patients who developed HBV-related liver cirrhosis had lower AST, ALT, GGT, and HBV-DNA levels. Liver synthetic function was even worse in cirrhotic patients reflected by lower serum albumin and cholinesterase than CHB patients (all P < 0.05). Serum FGF21 levels were significantly lower in cirrhotic patients (90.2 pg/ml [74.5–142.9]) compared with those in CHB patients (P < 0.001). CHB patients who developed ACLF had significantly higher indicators of inflammation and liver injury (CRP, AST, ALP, TBil, TBA, all P < 0.05). In those subjects with ACLF, serum FGF21 levels (407.1 pg/ml [141.8–640.0]) were significantly higher than those in CHB patients (P = 0.001). In CHB patients who developed HCC, the overall levels of serum α-fetoprotein had a trend to increase, but it was not statistically significant in our study (P = 0.287). In fact, 38% of these HCC patients showed normal α-fetoprotein levels. Notably, serum FGF21 levels in those patients exhibited a dramatic increase (398.6 pg/ml [244.0–827.6]) compared with CHB patients (P < 0.001) (Fig. 2).

**Table 2:** Anthropometric and biochemical parameters among CHB patients and CHB patients with advanced liver diseases.

Variables	CHB (n = 75)	CHB-Cirrhosis (n = 40)	CHB-ACLF (n = 17)	CHB-HCC (n = 9)
Age (year)	35.9 ± 11.7	47.6 ± 15.6^*^	41.4 ± 15.0	56.7 ± 8.7^*^
Male (n, %)	55 (73%)	29 (73%)	12 (71%)	7 (78%)
BMI (kg/m^2^)	22.3 ± 2.8	22.5 ± 2.4	23.1 ± 2.7	24.1 ± 2.8
AST (U/L)^†^	157.0 (79.0, 315.0)	50.5 (38.3, 138.0)^*^	405.0 (173.0, 879.0)^*^	65.0 (34.5, 117.5)^*^
ALT (U/L)^†^	320.0 (161.0, 754.0)	67.5 (33.0, 177.8)^*^	692.0 (268.0, 968.5)	57.0 (37.5, 122.5)^*^
GGT (U/L)^†^	84.0 (50.0, 166.0)	48.5 (25.8, 121.5)^*^	103.0 (59.5, 139.0)	147.0 (73.5, 232.5)
ALP (U/L)^†^	86.0 (66.0, 111.0)	92.0 (76.5, 126.5)	106.0 (91.5, 151.0)^*^	123.0 (92.5, 228.5)^*^
TBil (μmol/L)^†^	18.5 (13.3, 41.5)	24.6 (15.1, 48.3)	197.6 (98.3, 294.2)^*^	19.9 (9.7, 79.7)
TBA (μmol/L)^†^	16.2 (9.1, 53.9)	29.5 (10.8, 64.3)	169.1 (109.5, 208.2)^*^	6.9 (4.0, 86.6)
CRP (mg/L)^†^	2.0 (1.6, 4.4)	0.7 (0.3, 7.9)	9.6 (4.9, 16.2)^*^	9.7 (2.5, 11.4)
Cholinesterase (U/L)^†^	268.0 (186.0, 320.0)	164.0 (98.0, 251.5)^*^	140.0 (119.5, 173.5)^*^	208.0 (112.0, 353.0)
A/G^†^	1.60 (1.40, 1.80)	1.30 (0.88, 1.50)^*^	1.41 (1.12, 1.64)^*^	1.74 (1.22, 1.91)
Albumin (g/L)^†^	42.0 (38.0, 44.0)	35.0 (31.0, 40.3)^*^	34.0 (31.5, 38.5)^*^	42.0 (31.5, 45.0)
Total protein (g/L)^†^	67.0 (62.0, 71.0)	67.0 (62.5, 71.0)	61.0 (58.0, 65.0)^*^	66.0 (60.5, 72.5)
TC (mmol/L)^†^	4.0 (3.7, 4.5)	3.8 (3.2, 4.3)	2.6 (2.0, 3.3)^*^	4.2 (3.6, 5.6)
Triglyceride (mmol/L)^†^	1.1 (0.8, 1.4)	0.9 (0.8, 1.3)	1.4 (0.9, 1.9)^*^	1.1 (0.7, 1.2)
HDL-C (μmol/L)^†^	1.2 (0.6, 1.4)	1.3 (1.0, 1.4)	0.2 (0.1, 0.3)^*^	1.1 (0.2, 1.4)
LDL-C (μmol/L)^†^	2.2 (1.7, 2.7)	2.0 (1.8, 2.2)	1.2 (0.8, 1.7)^*^	3.2 (2.0, 3.5)
LgHBV-DNA (IU/ml)^†^	6.8 (5.4, 7.6)	4.2 (2.7, 5.7)^*^	6.5 (3.8, 7.6)	3.3 (1.0, 5.6)^*^
α-fetoprotein (μg/L)^†^	11.6 (5.2, 25.5)	14.5 (4.2, 46.0)	37.5 (18.7, 201.3)^*^	18.3 (4.8, 105.7)
FGF21 (pg/ml)^†^	142.8 (82.0, 265.3)	90.2 (74.5, 142.9)^*^	407.1 (141.8, 640.0)^*^	398.6 (224.0, 827.6)^*^

*P value < 0.05 vs. CHB ^†^Log-transformed before analysis.

ACLF, acute-on-chronic liver failure; A/G, albumin/globulin; ALP, alkaline phosphatase; ALT, alanine aminotransferase; AST, aspartate aminotransferase; BMI, body mass index; CRP, c-reactive protein; CHB, chronic hepatitis B; FGF21, fibroblast growth factor 21; GGT, gamma-glutamyl transpeptidase; HBV, hepatitis B virus; HCC, hepatocellular carcinoma; HDL-C, high-density lipoprotein cholesterol; LDL-C, low-density lipoprotein cholesterol; TBA, total bile acid; TBil, total bilirubin; TC, total Cholesterol.

### Serum FGF21 levels were positively correlated with the degree of acute liver injury and liver synthetic function

**Figure 3:**
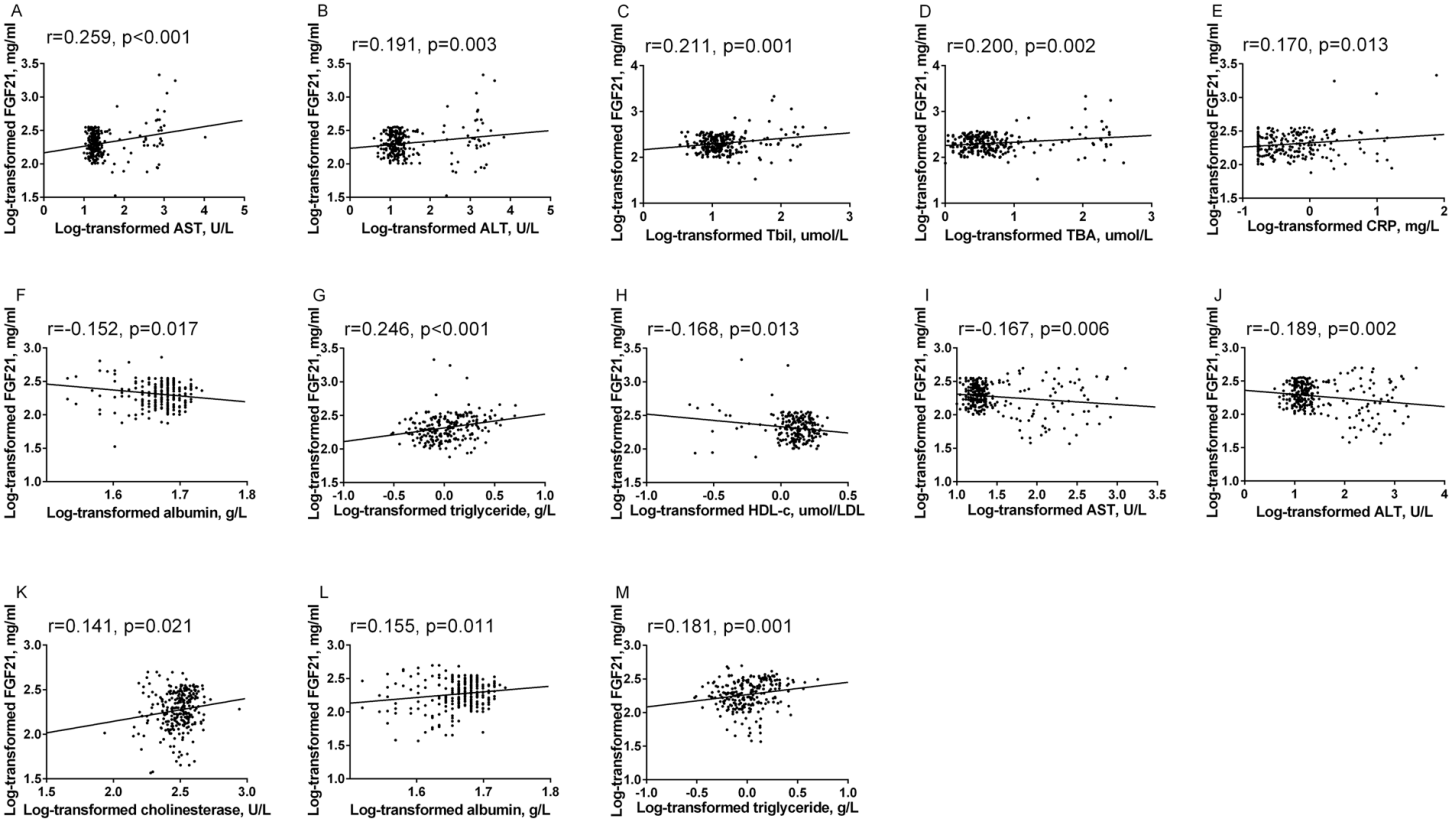
Relationship between serum FGF21 levels with other parameters in patients with AHB (**A–H**) and CHB (**I–M**).

Correlations between serum FGF21 levels and other biochemical indexes in AHB and CHB patients are shown in Table S1, and those with statistical significance are presented in dot plots (Fig. 3). In AHB patients, after adjustment for age and BMI, serum FGF21 levels were positively correlated with the elevation of AST, ALT, TBil, TBA and CRP (both P < 0.05). In CHB patients, serum FGF21 levels were positively associated with indicators of liver function including cholinesterase and albumin (both P < 0.05).

**Figure 4:**
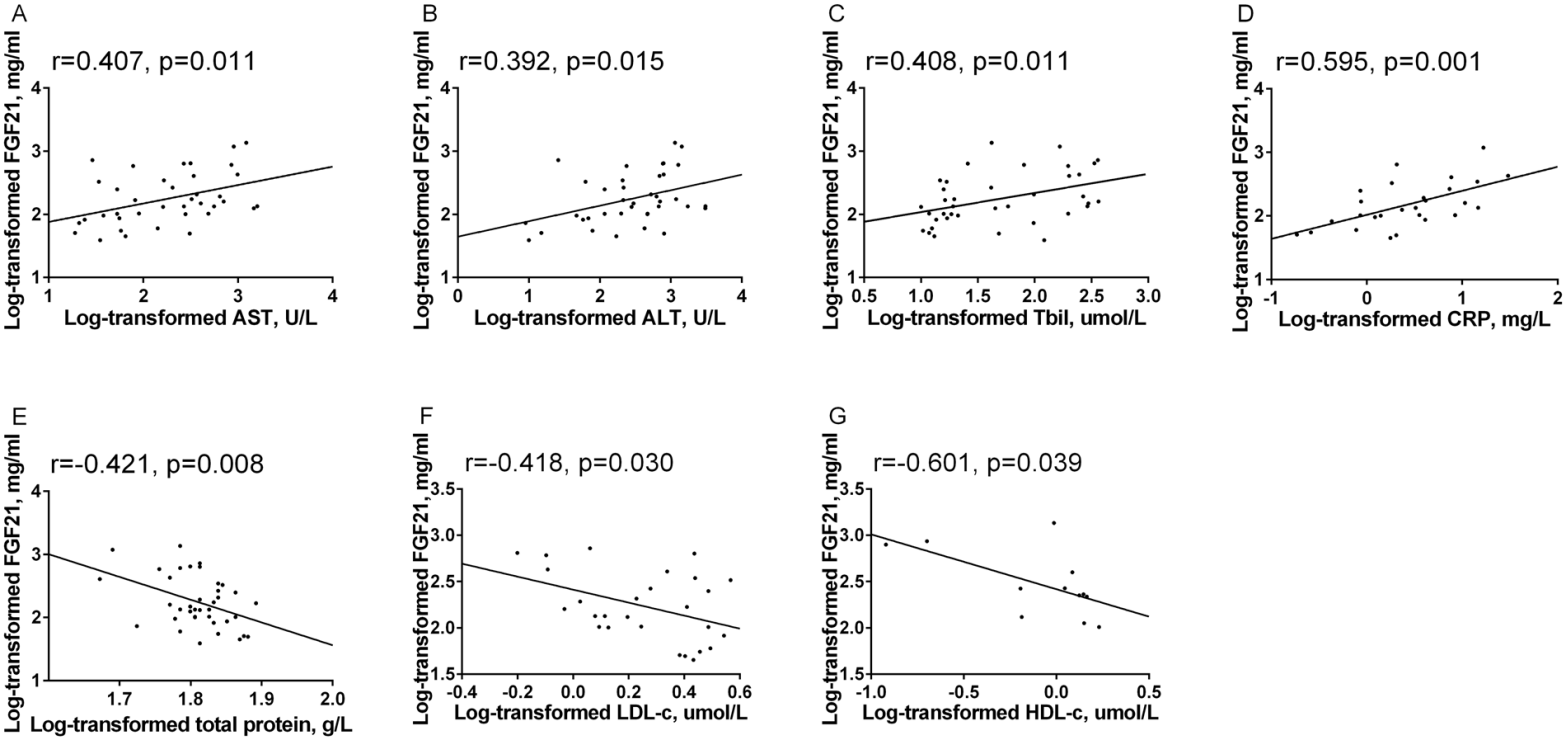
Relationship between serum FGF21 levels with other parameters in patients with CHB-ACLF (**A–F**) and CHB-HCC (**G**).

Correlations between serum FGF21 levels and other biochemical indexes in CHB patients with advanced liver diseases are shown in Table S2, and those with statistical significance are presented in dot plots (Fig. 4). In patients developed ACLF, serum FGF21 levels were positively correlated with indexes reflecting liver injury and inflammation including AST, ALT, TBil and CRP (all P < 0.05) after the adjustment for age and BMI. However, no significance was found between FGF21 and serum markers for liver fibrosis such as procollagen III n-terminal propeptide (P3NP), laminin, collagen type IV and hyaluronic acid in cirrhotic patients. Moreover, serum FGF21 levels were not correlated with α-fetoprotein in CHB patients who developed HCC. Notably, no significant associations were found between serum FGF21 levels and HBV-DNA load in each group no matter patients with AHB or CHB.

### Predictive value of FGF21 for HCC in CHB patients

**Figure 5:**
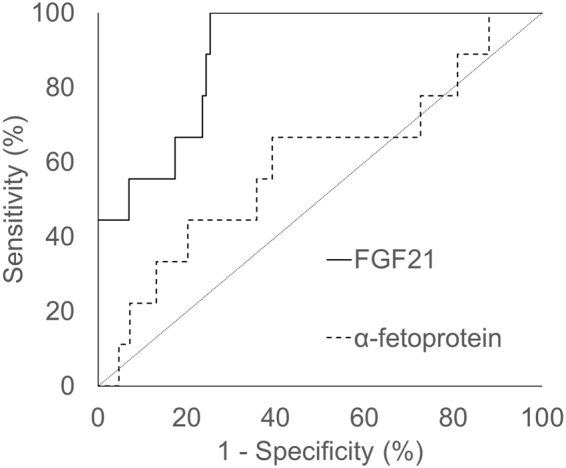
ROC curves for predicting HCC in CHB patients. FGF21, AUC = 0.892 (95% CI, 0.811–0.973); α-fetoprotein, AUC = 0.598 (95% CI, 0.388–0.807); FGF21 vs. α-fetoprotein, p = 0.01.

Serum FGF21 showed abnormal elevation in CHB patient who developed HCC, suggested that FGF21 elevation may provide information on HCC onset. To assess the predictive value of FGF21 for HCC, we generated the area under the receiver-operating characteristic (ROC) curve in all CHB patients except for those with ACLF. The result is shown in Fig. 5. For FGF21, the area under the curve (AUC) was 0.892 (95% CI 0.811–0.973, P < 0.001 compared with reference line). The AUC of FGF21 was larger than α-fetoprotein (0.598, 95%CI [0.388–0.807]), P = 0.01. The sensitivity, specificity, positive likelihood ratio, and negative likelihood ratio for predicting HCC with serum FGF21 are shown in Table 3.

**Table 3:** Sensitivity, specificity, positive likelihood ratio, and negative likelihood ratio for HCC prediction using serum FGF21 in CHB patients.

Cut-off of Serum FGF21 (pg/ml)	Sensitivity (%)	Specificity (%)	Youden index	Positive likelihood ratio	Negative likelihood ratio
200	100.0	74.8	1.748	3.968	0.000
225	77.8	76.5	1.543	3.311	0.290
250	66.7	80.9	1.476	3.492	0.412
275	55.6	82.6	1.382	3.195	0.538
300	55.6	84.3	1.399	3.541	0.527
325	55.6	86.1	1.417	4.000	0.516
350	55.6	89.6	1.452	5.346	0.496
375	55.6	93.0	1.486	7.943	0.477
400	44.4	93.0	1.374	6.343	0.598

Those result suggested that the increase of serum FGF21 was closely related to the active liver damage reflected by elevation of serum aminotransferases and CRP levels. Meanwhile, the decrease of serum FGF21 in patients with chronic HBV infection was mainly associated with the impaired liver synthetic function. Elevation of serum FGF21 in CHB patients may provide valuable information in the onset of HCC.

## Discussion

The study demonstrated for the first time the association of serum FGF21 levels with HBV infection. Serum FGF21 levels were significantly increased in AHB patients and then decreased rapidly to normal after antiviral and supportive treatment. To the contrary, serum FGF21 exhibited a marked decrease in patients with CHB, especially in cirrhotic patients and were related with liver synthetic function. Notably, with the occurrence of ACLF or HCC, serum FGF21 levels were increased dramatically in CHB patients. Serum FGF21 levels were found to reflect the degree of liver injury and impairment of synthetic function caused by HBV infection.

FGF21 is a stress-induced hormone in the liver. In our study, serum FGF21 levels were significantly higher in AHB patients and positively correlated with the elevation of serum aminotransferases and CRP, suggesting the consistency between serum FGF21 levels and the extent of liver damage. The release of mitochondrial enzymes from the liver is considered for evidence of hepatic necrosis^14^. In our study, FGF21 had a stronger association with AST, which might suggest that FGF21 regulated by mitochondrial stress and hepatic necrosis. After antiviral and supportive therapy, FGF21 decreased dramatically before the normalization of serum aminotransferases. These results suggest that FGF21 might be a quick detector reflecting conditions of liver stress and inflammation in AHB patients. ACLF is a serious consequence of the acute exacerbation of CHB in China^15^. Serum FGF21 levels in ACLF patients also showed similar changes as in AHB patients. Serum FGF21 levels in ACLF patients were increased remarkably and were positively correlated with CRP, aminotransferases, and other indicators of liver injury. These findings suggest that the severe endoplasmic reticulum (ER) stress and injury of hepatocytes in the progression of ACLF^16^ might lead to the increase of hepatic FGF21 expression.

HBV can interrupt protein folding and impair lipid metabolism in the host cell, which will initiate ER stress and induce oxidative stress^17–19^. Several studies have demonstrated that ER stress upregulates the expression of FGF21 in the liver^20–23^. IRE1α/XBP1 and PERK/eIF2alpha/ATF4 pathway of the unfolded protein response (UPR) can directly induce the expression of FGF21 by interacting with the response elements for XBP1 and ATF4 in the FGF21 promoter region^20–22^. FGF21 is also the target gene for C/EBP homologous protein (CHOP) as a downstream factor of ER stress^23^ and elevated FGF21 acts, in turn, to suppress the eIF2α-ATF4-CHOP signaling and alleviate ER stress-induced hepatic damage^20^. Besides ER stress, oxidative stress could also cause a dramatic elevation of both serum levels and hepatic expression of FGF21^9^. Increased FGF21 presents as a compensatory response for the liver to enhance antioxidant capacity and protects against hepatotoxicity^9^. Therefore, the elevation of FGF21 may partly be explained by ER stress and oxidative stress that participates in the progression of HBV infection and may exert a beneficial effect in counteracting stress-induced hepatic damage.

However, unlike in patients with acute liver injury, we noted that serum FGF21 levels were lower in CHB patients, although their serum aminotransferases and CRP were still higher than healthy subjects. In previous studies, the decrease of serum FGF21 was also observed under conditions of severe NAFLD and autoimmune diabetes, which resulted from impaired cell function caused by lipotoxicity, hepatic inflammation or autoimmune antibodies^24,25^. In our study, the drop of serum FGF21 was positively correlated with the decrease of cholinesterase and albumin in CHB patients, indicating a possible correlation between serum FGF21 and liver synthetic function. As the liver is the major source of FGF21, the impaired liver function may explain the decrease of FGF21 in CHB patients. In CHB patients who developed HBV-related liver cirrhosis, even lower serum FGF21 could be explained by worse liver function due to liver fibrosis^26^. Taken together, these observations support the idea that the decrease of FGF21 in CHB patients was mainly due to the poor synthetic function of the liver. However, FGF21 no longer responded to the prolonged ER stress in CHB patients. The underlying mechanism needs to be further studied.

Our result showed that serum FGF21 significantly increased in CHB patients who developed HCC, indicating that FGF21 could be induced by tumorigenesis in human. Previous study on human hepatic tissues demonstrates a high level of FGF21 expression in low-grade HCC foci area of well-differentiated cells, and also in tumor-adjacent and phenotypically normal liver area. Whereas the lost expression of FGF21 is found in high-grade HCC foci area of poorly differentiated tumor cells^8^. It has been reported that P53, an important tumor suppressor, negatively regulates the expression of FGF21 through binding to its promoter and mutant p53 leads to a drastic elevation of hepatic FGF21 in genetic-induced hepatocarcinogenesis mice model^8^. The elevation of FGF21 expression in hepatocyte may be a protective response, as evidence showed that forced expression of hepatic FGF21 in transgenic mice delays the initiation of chemical-induced hepatocarcinogenesis^27^, and loss of FGF21 plays an important role in HCC carcinogenetic transformation^28^.

Serum α-fetoprotein is the most widely used tumor marker for detecting HCC. However, elevated serum α-fetoprotein is only observed in 60–70% of HCC patients. Furthermore, nonspecific elevation of α-fetoprotein has been found in 15–58% of CHB patients and 11–47% of cirrhotic patients^29^. In our study, the abnormal elevation of serum FGF21 in HCC was more obvious, because the patients with CHB or liver cirrhosis had lower serum FGF21 levels. All the 9 patients with HCC had an FGF21 level over 200 pg/ml, whereas the median serum FGF21 levels in CHB and CHB-cirrhosis were 142.8 and 90.2 pg/ml, respectively. Although patients with ACLF also showed a significant elevation in serum FGF21, they had an obvious liver injury at the same time. Therefore, monitoring the elevation of serum FGF21 levels in CHB patients may provide valuable information about tumorigenesis, especially in those without obvious clinical signs of acute liver injury.

There are several limitations in our study. The first one is the lack of dynamic change of serum FGF21 since we only looked at one time point in each subject. Although we enrolled a subgroup of AHB patients after treatment, it is still unclear when is the turning point of serum FGF21 from a high level to low level. Serum FGF21 could change rapidly during acute liver injury according to our previous findings^9^. Secondly, the sample size of patients with HBV-related liver diseases was relatively small, especially for patients with HCC, which limited the further analysis to find out a diagnostic cutoff of serum FGF21 level. Thirdly, the age and BMI were not matched in disease groups, which may have some impact on the results. Besides, our result showed that, rather than a specific response to HBV infection, change of circulating FGF21 was more likely to be an indirect consequence induced by liver stress.

In conclusion, we present first clinical evidence revealing the diverse changes of serum FGF21 levels in HBV infected patients under different clinical conditions. Serum FGF21 levels were positively associated with serum aminotransferases and could reflect the degree of liver injury caused by HBV infection. However, serum FGF21 levels were decreased in CHB patients, especially in patients with HBV-related liver cirrhosis, due to impaired hepatic protein synthesis capacity. The abnormal elevation of FGF21 in CHB who developed HCC indicated the potential role of FGF21 as a biomarker in monitoring the tumorigenesis in CHB patients. Further studies are needed to clarify the cause-and-effect relationship between serum FGF21 and the progression HBV-related disease. More participants with advanced liver diseases should be involved in the study to make the results more reliable.

## Methods

### Subjects and study design

The study was cross-sectional designed. From October 2013 to June 2015, 186 HBV infected patients who were hospitalized in the Department of Infectious Diseases were observed. The number of cases in the hospital during the study period determined the sample size. Among all subjects, 33 had AHB, 75 had CHB, 40 had CHB-related liver cirrhosis, 17 had CHB-related ACLF, and 9 had CHB-related HCC. All subjects were interviewed to obtain their history of hepatitis, diabetes mellitus, hypertension, and cardiovascular diseases. Each patient was submitted to abdominal ultrasound or computed tomography (CT) scan. Fasting serum samples were drawn in the first morning after hospitalization. In a subgroup of AHB, the above examinations were conducted, and serum samples were drawn in these patients after receiving antiviral and supportive treatment for 11.8 ± 1.7 days. We also included age- and BMI-matched 200 healthy controls, who were recruited from our community-based epidemiological survey. Subjects with diabetes, obesity (BMI over 28) and cardiovascular diseases were not included due to their association with FGF21. The control subjects had no past or current viral hepatitis infection or other liver diseases. The study was approved by the ethics committee of the Shanghai Jiao Tong University Affiliated Sixth People’s Hospital, following the principles of the declaration of Helsinki. Written informed consent was obtained from all subjects. Authors removed personal identifier of the participants to protect confidential information.

### Diagnostic criteria

AHB was defined as patients with immunoglobulin M antibodies to the hepatitis B core antigen (IgM anti-HBc) and clinical signs or symptoms suggestive of acute hepatitis without a history of hepatitis B virus infection before the episode, and with the loss of hepatitis B surface antigen within six months after onset of acute hepatitis. CHB was defined as patients with positive HBsAg for more than six months, serum HBV DNA > 2000 IU/ml, ALT > 40U/L and excluding cirrhosis, liver tumor, and ACLF. CHB-related liver cirrhosis was defined as CHB patients with liver cirrhosis identified by ultrasound or CT. CHB-related ACLF was defined as CHB patients with acute hepatic insult manifesting as jaundice (serum bilirubin ≥ 85 μmol/L) and coagulopathy (international normalized ratio > 1.5), complicated within four weeks by ascites and/or encephalopathy^30^. CHB-related HCC was defined as CHB patients with space-occupying lesion in the liver discovered by ultrasound or CT.

### Anthropometric and biochemical measurements

BMI was calculated as the measured body weight in kilograms divided by the square of the measured height in meters. Biochemical indexes were measured on a Hitachi 7600 analyzer (Hitachi, Tokyo, Japan). Serum levels of total cholesterol (TC), triglyceride, high-density lipoprotein cholesterol (HDL-C) and low-density lipoprotein cholesterol (LDL-C) were determined enzymatically. ALT and AST were measured by ultra violet method. GGT was measured by the Szasz-Persijn method. HBV serologic markers were determined with a chemiluminescent microparticle immunoassay using the Abbott Architect immunoassay system (Abbott Laboratories, Abbott Park, IL, USA). The HBV DNA levels were measured by polymerase chain reaction using the Cobas Amplicor HBV monitor test (Roche Diagnostics, Mannheim, Germany), with a lower limit of quantification at 57 IU/ml. Concentrations of FGF21 in serum were quantified using the enzyme-linked immunosorbent assay kits (Antibody and Immunoassay services, The University of Hong Kong).

### Statistical analysis

All analysis was performed using SPSS 22 for Windows. Normality was determined by Kolmogorov-Smirnov test^31^. Normally distributed data were expressed as mean ± SD. Data that were not normally distributed were logarithmically transformed before analysis and expressed as median with interquartile range. The Student’s unpaired t-test was used for comparison between two groups. Correlations between parameters were determined by Pearson’s correlations and partial correlation. One-way ANOVA was used as appropriate for comparisons between groups, and multiple testing was corrected using Bonferroni correction. The accuracy of HCC prediction by serum FGF21 was evaluated using the area under the ROC curve. AUC was presented with 95% CI. In all statistical tests, P values less than 0.05 were considered significant.

### Data availability

The data are available from the corresponding author on reasonable request.

## Electronic supplementary material

Supplementary Information
