# Supplementary material for: Diverse Changes of Circulating Fibroblast Growth Factor 21 Levels in Hepatitis B Virus-Related Diseases
Source: Sci Rep. 2017 Nov 28;7:16482. doi: 10.1038/s41598-017-16312-6 (PMC5705770; doi:10.1038/s41598-017-16312-6)
Supplement: Supplementary file 1 — Supplementary Information [file 41598_2017_16312_MOESM1_ESM.pdf]

Supplementary Information

Diverse Changes of Circulating Fibroblast Growth Factor 21 Levels in Hepatitis B Virus-Related Diseases

Liang Wu[1,2¶], Qingchun Pan[3¶], Guangyu Wu[1,2], Lingling Qian[1,2], Jing Zhang[1,2], Lei Zhang[1,2], Qichen Fang[1], Guoqing Zang[3], Yudong Wang[4], George Lau[4,5,6], Huating Li[1*] and Weiping Jia[1*]

[1] Department of Endocrinology and Metabolism, Shanghai Jiao Tong University Affiliated Sixth People's Hospital; Shanghai Diabetes Institute; Shanghai Clinical Center for Diabetes, Shanghai, China

[2] Department of Medicine, Shanghai Jiao Tong University School of Medicine, Shanghai, China

[3] Department of Infectious Diseases, Shanghai Jiao Tong University Affiliated Sixth People's Hospital, Shanghai, China

[4] Division of Gastroenterology and Hepatology, Humanity and Health Medical Centre, Hong Kong S.A.R., China

[5] Second Liver Cirrhosis Diagnosis and Treatment Center, Beijing 302 Hospital, Beijing, China

[6] Institute of Translational Hepatology, Beijing 302 Hospital, Beijing, China

¶ These two authors contributed equally to the work.

***Corresponding author:**

Huating Li (M.D., Ph.D., E-mail: huarting99@hotmail.com) or Weiping Jia (M.D., Ph.D., E-mail: wpjia@sjtu.edu.cn). Address: 600 Yishan Road, Shanghai 200233, China, Tel: 86-21-64369181-8922, Fax: 86-21-64368031

Table S1. Correlations of serum FGF21 levels with biochemical parameters in AHB and CHB groups

Variables	AHB				CHB			
	Serum FGF21		Serum FGF21 (age & BMI adjusted)		Serum FGF21		Serum FGF21 (age & BMI adjusted)	
	r	p	r	p	r	p	r	p
Age	0.121	0.059	—	—	0.15	0.013	—	—
BMI	-0.048	0.456	—	—	0.033	0.586	—	—
AST	0.259	<0.001	**0.241**	**<0.001**	-0.167	0.006	**-0.14**	**0.024**
ALT	0.191	0.003	**0.177**	**0.006**	-0.189	0.002	**-0.159**	**0.010**
GGT	0.135	0.035	0.116	0.074	-0.138	0.024	-0.119	0.054
ALP	0.114	0.075	0.1	0.123	-0.029	0.638	-0.007	0.915
TBil	0.211	0.001	**0.191**	**0.003**	0.051	0.403	0.066	0.288
TBA	0.2	0.002	**0.186**	**0.004**	-0.069	0.261	-0.041	0.508
CRP	0.17	0.013	**0.178**	**0.01**	-0.017	0.801	-0.011	0.874
Cholinesterase	0.045	0.483	0.044	0.498	0.141	0.021	**0.125**	**0.044**
A/G	-0.085	0.185	-0.093	0.15	0.086	0.161	0.08	0.199
Albumin	-0.152	0.017	**-0.139**	**0.032**	0.155	0.011	**0.145**	**0.019**
Total protein	-0.062	0.332	-0.037	0.57	0.107	0.081	0.101	0.106
TC	0.034	0.611	0.027	0.689	0.107	0.093	0.107	0.094
Triglyceride	0.246	<0.001	**0.245**	**<0.001**	0.181	0.004	**0.197**	**0.002**
HDL-C	-0.168	0.013	**-0.161**	**0.017**	-0.058	0.385	-0.081	0.227
LDL-C	-0.078	0.250	-0.075	0.27	0.119	0.071	0.125	0.06
LgHBV-DNA	0.251	0.109	0.238	0.155	-0.045	0.716	0.016	0.904

All data had been log-transformed before analysis except for age and BMI.

A/G, albumin/globulin; AHB, acute hepatitis B; ALP, alkaline phosphatase; ALT, alanine aminotransferase; AST, aspartate aminotransferase; CRP, c-reactive protein; CHB, chronic hepatitis B; FGF21, fibroblast growth factor 21; GGT, gamma-glutamyl transpeptidase; HBV, hepatitis B virus; HDL-C, high-density lipoprotein

cholesterol; LDL-C, low-density lipoprotein cholesterol; TBA, total bile acid; TBil, total bilirubin; TC, total Cholesterol.

Table S2. Correlations of serum FGF21 levels with biochemical parameters in CHB patients with advanced liver diseases

Variables	CHB-Cirrhosis				CHB-ACLF				CHB-HCC			
	Serum FGF21		Serum FGF21 (age & BMI adjusted)		Serum FGF21		Serum FGF21 (age & BMI adjusted)		Serum FGF21		Serum FGF21 (age & BMI adjusted)	
	r	p	r	p	r	p	r	p	r	p	r	p
Age	0.103	0.365	—	—	0.074	0.661	—	—	0.186	0.461	—	—
BMI	0.162	0.202	—	—	-0.022	0.899	—	—	0.028	0.914	—	—
AST	0.066	0.571	0.100	0.445	0.407	0.011	**0.430**	**0.013**	0.252	0.312	0.237	0.396
ALT	0.005	0.964	0.041	0.754	0.392	0.015	**0.457**	**0.007**	0.119	0.637	0.119	0.672
GGT	0.09	0.435	0.138	0.293	0.188	0.258	0.202	0.260	0.365	0.136	0.363	0.183
ALP	0.045	0.697	0.053	0.689	0.173	0.3	0.171	0.341	0.33	0.18	0.354	0.196
TBil	0.026	0.822	0.035	0.791	0.408	0.011	**0.41**	**0.018**	0.451	0.06	0.494	0.062
TBA	0.084	0.468	0.103	0.433	0.283	0.085	0.277	0.118	0.468	0.05	0.484	0.068
CRP	0.231	0.288	0.338	0.145	0.595	0.001	**0.593**	**0.002**	0.568	0.111	0.559	0.192
Cholinesterase	-0.014	0.901	-0.019	0.886	-0.211	0.202	-0.201	0.263	-0.381	0.119	-0.411	0.128
A/G	0.02	0.862	0.042	0.749	-0.06	0.719	-0.05	0.781	-0.018	0.943	0.041	0.883
Albumin	0.078	0.499	0.112	0.396	-0.344	0.035	-0.338	0.054	-0.159	0.527	-0.165	0.557
Total protein	0.102	0.377	0.116	0.376	-0.421	0.008	**-0.417**	**0.016**	-0.186	0.461	-0.247	0.376
TC	-0.031	0.838	-0.002	0.990	-0.356	0.039	-0.349	0.059	-0.253	0.405	-0.372	0.29
Triglyceride	-0.018	0.905	-0.009	0.955	-0.108	0.563	-0.120	0.544	-0.193	0.527	-0.13	0.72
HDL-C	-0.064	0.725	-0.100	0.625	-0.339	0.083	-0.345	0.098	-0.601	0.039	-0.824	0.006
LDL-C	0.125	0.489	0.145	0.479	-0.418	0.03	**-0.419**	**0.042**	-0.176	0.585	-0.29	0.449
LgHBV-DNA	-0.225	0.078	-0.214	0.154	0.028	0.88	0.061	0.758	-0.065	0.833	-0.101	0.78
α-fetoprotein	-0.026	0.86	-0.033	0.829	0.07	0.713	0.086	0.662	0.36	0.143	0.36	0.187
P3NP	0.085	0.619	0.128	0.478	-0.035	0.887	-0.043	0.87	0.07	0.87	0.038	0.951
Laminin	0.017	0.921	0.024	0.894	-0.179	0.463	-0.179	0.491	-0.261	0.533	-0.348	0.566

Collagen type IV	0.006	0.971	0.029	0.871	-0.133	0.588	-0.145	0.58	0.039	0.927	0.027	0.966
Hyaluronic acid	0.104	0.539	0.12	0.507	0.16	0.513	0.147	0.575	0.104	0.807	0.067	0.914

All data had been log-transformed before analysis except for age and BMI.

A/G, albumin/globulin; ACLF, acute-on-chronic liver failure; ALP, alkaline phosphatase; ALT, alanine aminotransferase; AST, aspartate aminotransferase; CRP, c-reactive protein; CHB, chronic hepatitis B; FGF21, fibroblast growth factor 21; GGT, gamma-glutamyl transpeptidase; HBV, hepatitis B virus; HCC, hepatocellular carcinoma; HDL-C, high-density lipoprotein cholesterol; LDL-C, low-density lipoprotein cholesterol; P3NP, procollagen III n-terminal propeptide; TBA, total bile acid; TBil, total bilirubin; TC, total Cholesterol.
